# Experiences of a multiple intervention trial to increase maternity care access in rural Tanzania: Focus group findings with women, nurses and community health workers

**DOI:** 10.1177/1745506520969617

**Published:** 2020-11-04

**Authors:** Gail Webber, Bwire Chirangi, Nyamusi Magatti

**Affiliations:** 1 Department of Family Medicine, Bruyere Research Institute, University of Ottawa, Ottawa, ON, Canada; 2 Shirati KMT Hospital, Mara, Tanzania

**Keywords:** birth kits, community health workers, facility births, maternity care, maternal mortality, m-health, misoprostol, Tanzania

## Abstract

**Objectives:**

In order to improve maternal health and women’s access to maternity care services in Rorya District, Mara, Northern Tanzania, we introduced several interventions across the district from 2018 to 2019. The interventions were workshops with nurses to encourage respectful care of women and transportation subsidies for women to reach the health facilities for delivery. In addition, we trained community health workers to educate couples about safe birthing options using m-health applications, to collaborate with nurses to distribute clean birth kits with misoprostol and to hold village meetings to shift community norms. This article reports on the experiences of women, community health workers and nurses during the study.

**Methods:**

Focus group discussions were conducted with a convenience sample of these groups to understand the successes and challenges of the interventions.

**Results:**

The workshops with nurses to encourage respectful maternity care and the birth kits with misoprostol were appreciated by all and were an incentive for women to seek health services. While the m-health applications were innovative, the system required significant oversight and a stable network. The village meetings demonstrated some success and should be expanded. Travel subsidies were problematic to implement and only helpful to the minority who received them.

**Conclusion:**

Multiple intervention strategies are needed to help women access maternity care services in rural locations and should be designed to meet needs within the local context. In Rorya District, access to quality health care was improved through training nurses to provide respectful care and using community health workers to educate the population about safe birthing practices and to provide women with clean birth kits. Despite the current limitations of m-health, there is much potential for development. Finding solutions to women’s need for transport is a particular challenge and will likely require innovative community-based approaches.

## Background

Maternal health during pregnancy and delivery is directly related to access to quality health care services. Skilled attendance at delivery is critical for prevention and treatment of conditions leading to maternal demise such as haemorrhage, sepsis and hypertensive disorders of pregnancy. Despite the emphasis on maternal mortality reduction in the millennium development goals (MDGs), the maternal mortality ratio in sub-Saharan Africa remains unacceptably high at 545 per 100,000 as of 2015.^1^ The sustainable development goals which followed the MDGs have an impressive target to reduce the global maternal mortality ratio to less than 70 per 100,000 by 2030. Unfortunately, however, with only 59% of women being cared for by skilled birth attendants in sub-Saharan Africa (as of 2018), this goal will be challenging to achieve.^2^ For many women in rural Africa, the barriers to access maternal health services are very difficult to overcome. In the Lake Zone and Western regions of Tanzania, the facility birth rate was only 40%–50% according to the most recent 2016 District Health Survey; Mara Region ranked among the bottom third of regions in the country for facility births.^3^

To explain the context in which women seek maternity care, Thaddeus and Maine^4^ developed the ‘Three Delays Model’, a framework of the barriers to accessing health care services at the time of delivery. The framework describes the crucial delays in *making a decision* to access care, delays in *reaching the health facility* and delays in *receiving care at the health facility.* To explore the key factors contributing to the three delays in the rural context of Tanzania, we held a participatory consultation with community members and policy makers in Rorya District of Mara Region in 2015.^5^ These participants identified the following priorities for strengthening maternal health: to improve the transportation for women to access the health facility, increase the availability of supplies in the health facilities, address negative healthcare provider (HCP) attitudes towards women and to increase the number of skilled HCPs. Policy makers also prioritized improved health education of women, improved access to health facilities (reducing distances and increasing hours of operation) and increased power in decision-making for women.

**Figure 1.**
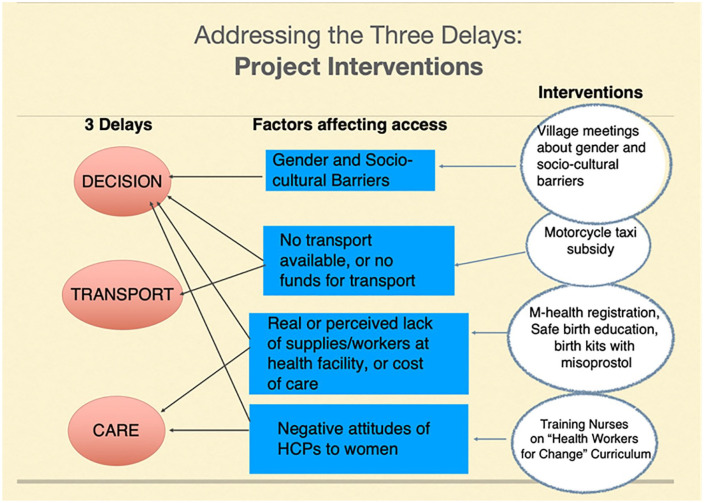
The project interventions to address the three delays and barriers to access.

To address these barriers, we developed a multiple intervention project focussing on four major barriers to access for this population of women in Rorya District. These barriers were defined as follows: (1) gender and cultural barriers, (2) lack of available transport or funds for transport, (3) real or perceived lack of supplies and workers at the health facility and cost of facility care and (4) negative attitudes of HCPs towards women. As illustrated in Figure 1, each of these factors impacts on one or more of the three delays. We designed four complementary interventions to address the barriers to care. First, we trained nurses working in maternity care on the ‘Health Workers for Change’ curriculum.^6^ The purpose of this training was to encourage them to provide more respectful care to women. During the evaluation of the workshops, the nurses reported that they found this training to be helpful at sensitizing them to women’s needs.^7^ A second key intervention was training community health workers (CHWs) to register pregnant women using mobile phone applications and educating them about receiving health care services. In addition, the CHWs worked with nurses to distribute clean birth kits with misoprostol to be taken for postpartum haemorrhage prevention when oxytocin was not available. The women were encouraged to bring the birth kits to the health facility at the time of their delivery, thus bypassing the need to purchase supplies if the health facility was lacking in them. The CHWs were also expected to hold community meetings to educate women and family members about the importance of supporting women to access medical care during the pregnancies and deliveries. Finally, transportation subsidies were provided for women who lived more than 2 km from the health facility for women to access local motorcycle taxis. The purpose of this article is to explore the experiences of women, CHWs and nurses who were involved in this project.

## Methods

This article reports on the qualitative findings from the third phase of the EACH WOMAN Health project (‘Enhancing all Community Health workers on Maternal and Newborn Health’) conducted in Rorya District of Mara Region, Tanzania, from 2015 to 2019. Phase one consisted of a community and policy maker consultation to identify the barriers for women to access maternity services.^5^ The second phase included a training programme for maternity nurses focused on providing respectful care to women.^6^ The third and final phases were a multiple baseline trial in which interventions to improve access to maternity care were introduced to the different divisions of Rorya District sequentially after baseline data collection. Focus group discussions (FGDs) were held with women, nurses and CHWs who were involved in the trial, during the later months of the project in 2018 and 2019. We obtained ethical approval from the Ottawa Health Science Network Research Ethics Board, Bruyere Research Ethics Board (in Ottawa, Canada) and the Ethics Board of the National Institutes of Medical Research, Tanzania (Dar es Salaam). The FGDs were held in locations across the district, usually close to a local health facility. We recruited a convenience sample of women, nurses and CHWs from among those who were involved in the project. All participants read (or had read to them) a consent form and signed the form. Confidentiality of responses was assured. Four focus groups were held with women, five with nurses and four with CHWs. The total number of participants was 112 (31 women, 38 nurses and 43 CHWs). The FGDs took place in the local language of Swahili. They were recorded and then transcribed and translated to English. Nvivo software (version 12.6.0) was used to assist with the thematic analysis. The themes focussed on experiences of the participants with each of the interventions in the project. The themes were determined through iterative readings of the transcripts and representative quotations of the participants are presented.

## Results

The purpose of the FGDs was to determine the experiences of the women, nurses and CHWs during the project interventions to assess the strengths and challenges of the project and make recommendations for future scale-up. The results are presented structured around the four interventions: CHW-led village meetings for health education, (2) transportation subsidy, (3) CHW registration of women using m-health applications and distribution of birth kits with misoprostol and (4) training HCPs on respectful maternity care.

### CHW-led village meetings

To address the cultural and gender barriers to accessing health care services, CHWs were instructed to hold village meetings to introduce the project to the community. The purpose of the meetings was to educate them about the importance of women receiving skilled attendance for antenatal care, delivery and postpartum care. Not all CHWs were able to do this, as coordinating a meeting required collaboration with village leaders; however, they found other ways to meet with women.

I was not able to conduct these meetings, because my village chairman was not able to call meetings and even to give me chance to do so. What I did was to give a seminar to each women at their homes, or when they come to clinic and are together, this is the time when I meet them and give them health education. (CHW, Shirati FGD)

While individual or group health education with women was important, the benefit of village meetings was the opportunity to reach a larger audience, including male partners and other community members. When the CHWs visited women at home or in the clinic setting, men were not often present. Support during health care services for women is not a traditionally acceptable role for men. In contrast, village meetings are a common place for men to gather.

CHWs had the village meeting. The main aim was to visit the women in their respective areas and inform them about the project, what is being done. The meeting has helped so much because you meet their husbands at the meeting; so meeting is jointly with father and mother, men are being told the importance of attending the clinic, most of them understand. (Nurse, Nyarombo FGD)

Thus, another potential benefit of village meetings over individual health counselling is providing a forum for addressing gender and cultural barriers and taboos in a group setting. Future projects should invest more time engaging community leaders to support the CHWs to provide village meetings and training CHWs in how to challenge community norms.

### Transportation

Transportation to the health facility for the mother was the most challenging barrier to address in this impoverished population. After considerable discussion, we chose to provide our transportation subsidy to the nurses to pay out to motorcycle taxi drivers (known as ‘bodabodas’) when they transported women to the health facility from at least 2 km away.

This is my third delivery, we are very thankful because of what you have done for our to us, We are getting transport fare, before the research we got nothing. We were giving birth on the road. When you call the bodaboda he doesn’t come because he knows you don’t have money. But now whenever you call bodaboda he comes immediately because he knows he will get paid. (Woman, Kowak FGD)

While many appreciated the free service, for others, the transportation subsidy was not accessible. The strategy of having nurses control the funds for transportation stipends was not always successful, as the next three quotations illustrate.

I had a challenge when I ask the bodaboda to take me to the health facility and I pay him and then claim for this money to be refunded, it became difficult because they need the telephone number and plate number of the bodaboda which I don’t have at the moment. (Woman, Kinesi FGD)

Most women are motivated to attend clinic since the starting of the project. But the challenge I see is sometimes the transport fare is not available or finished, so if I tell the woman she doesn’t agree that it is true, she thinks that I have used the money. (Nurse, Utegi FGD)

Sometimes when the pregnant mothers go to the facility to deliver and there is no money they come back to CHW to demand their money, so make sure there is always money at the facility. (CHW, Tatwe FGD)

The funds were only intended to pay for the woman herself; however, understandably, women preferred not to be alone at the health facility. This created conflict when payment for the ride was due, as the cost of accompanying persons was not included in the subsidy.

Another challenge is that bodaboda comes in with three people including the pregnant mother, and he wants to be paid for three which is nine thousand instead of three thousand so sometimes it brings strong argument and misunderstanding. (Nurse, Kowak FGD)

Finally, for those who delivered before reaching the institution, the funds did not apply.

Transportation money wasn’t given to me simply because I couldn’t make it to the health facility due to the fact that I delivered on my way to the health facility unfortunately. (Woman, Utegi FGD)

Thus, while transportation is a significant barrier for women to access care in this rural district and motorcycle transport is the only available option, subsidizing the rides created multiple challenges. Who should be responsible for holding the funds? What happens when the funds are no longer available? Who gets transported–only the mother or is she permitted support people? What are the transport options for women who live closer? Even 2 km is a great distance to walk when you are in active labour. Providing the funds at the institution is neither feasible nor sustainable and increases the workload of the nurse. Future projects should seek community-based solutions acceptable and accessible to the whole population.

### m-health registration and birth kit distribution

This intervention served to address several identified barriers to women accessing health care services: lack of education about safe births, real or perceived lack of supplies at the health facility and informal costs of care. Prior to the project, many women were delivering with local traditional birth attendants (TBAs) as local customs dictated that home births were superior. The CHWs provided health education about the danger signs in pregnancy and the importance of seeking care with skilled HCPs for antenatal care and delivery. The nurses appreciated the work of these committed CHWs.

CHWs are working very hard, because they are visiting home by home. If a pregnant women is having danger signs he/she refers this woman to the health facility where she gets care. This is very good work. We should continue to motivate the CHWs so that they continue to work well. (Nurse, Kinesi FGD)

A key component of this intervention was provision of a phone and airtime to each CHW with the m-health app installed on the phone. The use of the m-health app was novel in this population of CHWs and not without its challenges. Several of the CHWs were unfamiliar with the use of phone applications and required considerable support from the research team. Charging the phones was problematic: while we provided solar chargers because access to electricity was not always available, the CHWs complained that the solar chargers were slow to charge the phones. Finally, the network created large challenges. The CHWs found that when they submitted their registrations, it took time to receive the identification number for the women. On occasion, CHWs would receive multiple numbers after submission, likely from multiple attempts to submit. This created great confusion for both the CHWs and the research team (and subsequent problems in cleaning the data at the end of the project). Despite these challenges, the CHWs appreciated that the phones simplified their work. The m-health app reduced the CHWs need to travel to submit reports. In addition, the phones provided status to the CHWs and had added benefits for health education, with reminders of key information to share with the women:

CHWs using phones has helped them so much to empower them mentally, and provide self confidence, and respect by community members. . . CHWs are visiting women in their respective villages and registering them by using phones. CHWs are able to give the information which is in the phone. They are also able to tell information concerning danger signs easily by using the phone. Through this women can be helped to identify danger signs. (Nurse, Nyarombo FGD)

The health facilities often lacked crucial supplies such as gloves, cord clamps and medication. As a result, families were commonly asked to purchase these supplies. Training CHWs to collaborate with nurses to provide birth kits with the needed supplies alleviated these barriers to care. The birth kits contained soap for the woman to wash, two pairs of gloves, a pad for delivery (known as a mackintosh) and a postpartum pad, two cord clamps, a surgical blade to cut the cord and 600 mcg of misoprostol for the woman to take after delivery for postpartum haemorrhage prevention. The CHWs were in a good position to identify and recruit pregnant women early in their pregnancies, as they were trusted members of the villages themselves.

This intervention was appreciated by all three groups of participants and led to increased healthcare-seeking behaviour.

I was given a birth kit by the community health worker and they explained to me everything inside the birth kit: soap, maternity pads, mackintosh, blade, gloves, and the drug which prevents bleeding. They also explained to me how to use them. We went with my husband to the clinic. I was very happy to get health education also. The services we are getting have improved. (Woman, Kowak FGD)

The way I see the development of this project, before pregnant mothers used to deliver at the traditional birth attendants’ home, but today there is a big change, most of them go to the clinic. Today pregnant mothers are searching for CHWs so that they can be registered. Pregnant mothers go to the clinic even when are only two months pregnant so they can be registered and enjoy birth kit services during delivery. (CHW, Utegi FGD)

The provision of birth kits to mothers prior to delivery was not universally accepted by all HCPs, however. Some were concerned that if women had the materials they needed to deliver, they would then choose to avoid the health facility in favour of a home birth.

The process of giving CHWs birth kits to distribute to pregnant women is not good, because if the woman is given a birth kit she can decide to go anywhere she likes to go to deliver. We have been providing birth kits to CHWs to give pregnant women, sometimes it happens that she doesn’t come to have birth at the healthy facility. Then she will tell you that I went to give birth at the traditional birth attendants’. If you ask her about the birth kit she will tell you I have already delivered because you gave me a birth kit. According to my opinion, I would like the birth kits to remain at the health facilities, so the nurse can provide them for the women. (Nurse, Nyarombo FGD)

Another point of contention was the contents of the birth kits. Many of the nurses would have preferred if the kits could contain more pairs of gloves, cotton wool and a larger surgical blade. The women requested other items such as clothing for the infant, diapers and more pads. All of the women were very pleased with the bag that the birth kit came in as they used it after delivery for carrying their infant’s clothing. Stronger instruction on keeping the bag intact and free of other items prior to delivery was needed in some cases, as a few women arrived for delivery without the original contents.

Overall, the registration and education of women using m-health apps and distribution of birth kits were a positive intervention to address the needs for education and access to supplies at the time of delivery. Future projects using m-health apps with rural CHWs will require greater investments in supervision and maintenance of the m-health database.

### Respectful maternity care training: ‘Health Workers for Change’

Improving the attitudes of the HCPs towards their women patients was one of the key priorities determined by the 2015 consultation with community members and policy makers.^5^ After the ‘Health Workers for Change’ workshops with HCPs, women noted a significant change in how they were treated by the nurses. Women reported better engagement with the HCPs – they were both more available and more supportive. Women did not have to wait as long to receive care, and they found that the nurses were kinder. The woman quoted below recalled her most recent experience in the health facility much more favourably than previous experiences.

Today health services are perfect and up to date; we are given a warm welcome at the health facility. Before that services were very poor and discouraging as the nurses used harsh language. (Woman, Tatwe FGD)

The CHWs reported that they noted increasing numbers of women attending the facilities for care and that mothers were more satisfied with the care they received from the nurses.

At my health facility everything is preceding well, they get pregnant women and the feedback that we get from the mothers is that they receive good care from the nurses when they are there. (CHW, Utegi FGD)

The nurses also reflected on their change in attitude after the training. The culture had shifted: it was no longer acceptable to express frustration and anger towards the patient. Women were treated with more respect. The increase in number of women attending the health facility was attributed by some to be due to the improvement in their attitudes.

For myself [the training] has helped so much. For example, sometimes the women may come when I am very tired. The care which I will give, she will feel that it is not satisfactory; but what I got from training is that in spite of tiredness I should try my level best to provide quality care to this woman and she should show that she is satisfied with my care. (Nurse, Nyarombo FGD)

Now we have good behaviour and the number of women attending our health center is high. Before, deliveries were 19 to 20 per month, but now 50 women are giving birth per month [at the health facility]. (Nurse, Utegi FGD)

The workshops for nurses on attitudes towards their women patients were a worthwhile investment that paid off for all participants. The women received better care, the CHWs found it easier to encourage women to attend the health care facility and the nurses were more satisfied with the care they gave and the increased numbers of women seeking their services. In order to sustain these changes, refresher courses to maintain their positive attitudes towards their patients were suggested by the participating nurses. This would need to be budgeted for in future projects.

## Discussion

The barriers to access health facilities for pregnant women demonstrated in our study are consistent with what has been documented in the literature. In a qualitative review of the facilitators and barriers to accessing health care services for delivery, the common barriers were cost, influence of others on birthing decisions, lack of plan for childbirth, fear of HIV testing, lack of affordable transportation options, health care policies, perception of risk, perceived quality of care, medicalization of childbirth at the health care facilities, influence of culture and tradition and the perceived easier logistics of home birth.^8^ A review of mixed methods studies on access to emergency obstetric care at health facilities in sub-Saharan Africa found an even more extensive list of barriers impacting on all three delays, including many deficiencies in the quality of care at health care facilities.^9^

### The first delay: decision

In this group of women, the decision to go to the health facility was not women’s alone. Husbands and mothers-in-law have significant power in choosing if women should leave home to have a health facility birth, as this can incur more costs than staying at home or delivering with a local TBA. Men often under-estimate the risk of childbirth to women.^10^ Thus, health education must be directed towards the family members and the community, if cultural and gender norms are to be shifted. Village meetings are one method to do this; men attending the village meetings did appear to be more engaged in their partners’ care. Other methods used in rural Tanzania to increase male involvement and support for facility birth and shared decision-making include CHW training of men about the risk of childbirth and need for a facility delivery.^11^ While involving men more in women’s health care decisions, care must be made to ensure her wishes are considered.^12^ In our experience, the gender of the CHW may be important in male education; men were more likely to heed the views of male CHWs than females.

Other factors affecting the decision to go to the health facility include the cost and availability of transport and the perceived care at the health facility. Solutions to these issues will be reviewed under the second and third delays.

### The second delay: transport

Solutions to transport need to be community-based. Our attempts to provide subsidies through the health facility demonstrated that this solution has flaws and is not sustainable. Funds were not available to all who needed them, and there was confusion about who was eligible for free transport. Previous experience in the region with motorcycle ambulances was disappointing, as the ambulances did not work well and were ultimately abandoned. Providing vouchers directly to women has not been entirely successful in Kenya, as the high cost of transport prevented use of the voucher for some women.^13^ Encouraging families to save for transport at the time of delivery or contributing to a community fund to pay for transport are possible solutions being explored in an ongoing project in Mara Region (United States Agency for International Development (USAID) funded project, personal communication, Dr Florian Tinuga, Mara Regional Medical Officer). Community-based solutions are likely to be more sustainable in the long term and should be the focus of future research.

### The third delay: care at the health facility

Our interventions to engage women in receiving care at the health facility included using CHWs to register and educate them using their m-health phone applications. While the CHWs in our study had significant challenges with the m-health applications, they did recognize the benefits of the application for health education, data collection and convenient submission of results. A review of CHW use of m-health applications documents that there are many pilot studies of m-health use for maternal health projects among others; however, scale-up has rarely occurred.^14^ Indeed, within Tanzania, there have been several projects demonstrating the success of m-health applications;^15–17^ however, the Ministry of Health has yet to implement a national programme. Rwanda’s Ministry of Health has developed an extensive programme of CHWs using m-health applications to send rapid-SMS if women need care urgently. A qualitative study on the CHWs’ views of the programme show similar challenges to our project. More CHW training and closer supervision is required, in addition to better solutions for charging the phones.^18^ Clearly, m-health shows great potential; however, the implementation requires large investments of time and resources for supervision and development of the application that are not always available in already stressed health care systems.

Provision of birth kits with misoprostol directly to women served to reassure them that the supplies needed for birth were available, and thus a visit to the health facility was not likely to incur extra cost. The birth kits also relieved the nurses of the stress of requesting the families to purchase supplies. As the contents of the kits are usually provided at the health facilities, the cost of birth kits should be borne by the health care system. Mara Regional Medical Office is currently considering including birth kits in their health facility budgets (personal communication, Dr Florian Tinuga, Mara Regional Medical Officer). Ensuring a consistent supply will be important. CHW distribution of the birth kits remains a contentious issue for a minority of policy makers and HCPs. While some feel allowing women to have birth kits before reaching the health care facility will permit women to choose home births, there is no evidence that home births are increasing with the provision of birth kits. Indeed, in our study in the neighbouring districts in Mara Region, birth kits appeared to contribute to a rise in health facility births.^19^ Women who cannot reach the health facility due to precipitous delivery or those who prefer to avoid a facility birth are the most vulnerable to postpartum haemorrhage and sepsis. It is our contention that if women do not receive a birth kit from the nurse by 36 weeks of gestation, a CHW should be dispatched to educate the women and provide the kits.

Disrespectful care of pregnant women has been documented as a significant barrier to care by numerous studies and can vary from harsh comments and abuse, to lack of privacy, to delays in receiving appropriate care.^20^ We have demonstrated that nurses’ negative attitudes towards women in our population can be improved through the ‘Health Workers for Change’ programme.^7^ This programme can be implemented relatively inexpensively in a 6- to 9-h training programme, though refresher training is also advisable. Changing the culture of how women are treated is worthy of this investment.

## Limitations

There are several limitations to this qualitative study of our multiple intervention trial. Social desirability bias may cause the participants to provide the researcher with the answers they think the researchers want to hear. To avoid this, we deliberately asked probing questions to ensure participants were honest in their responses. We are reassured that several participants spoke of their dissatisfaction with components of the project such as the transport subsidy. We attempted to include women, nurses and CHWs from geographically diverse communities within Rorya District in order to ensure the generalizability of the data; however, it is possible that some views were not represented in our sample. This study is unique in that it provides contextual perspectives on the experiences of women, nurses and CHWs involved in specific interventions to increase women’s access to health care services.

## Conclusion

The barriers to health care services at the time of childbirth for women living in rural Africa are many. This study reports on the third phase of a research project to address these issues. The choice of interventions was determined by a community and policy maker participatory consultation. Training nurses to provide respectful care was the second phase of the research, in the preparation for the trial. The other interventions in the trial included CHW-led village meetings, registration of pregnant women and health education by CHWs using m-health apps, distribution of birth kits with misoprostol and transportation subsidies. The successful interventions which should be considered for scale-up include the participatory ‘Health Workers for Change’ curriculum to encourage respectful care of women through self-reflection and the distribution of birth kits with misoprostol. m-health has great potential; however, more investment in supervision and management of the database is needed. In addition, in our rural location, the network was not reliable enough during the study period. Solutions for transportation should not be facility-based, but instead require community consultation and development. Despite the challenges, women appreciated the efforts to help them achieve safe delivery. These efforts need to be built upon to ensure all women have access to life-saving skilled attendants and supplies at birth.

We have got help from the project, before project we were not motivated, and most women had birth at home. But during the project we are now motivated to go to the health facilities to have birth. When the project started we are more motivated because we are now getting transport to the health facility, we also given birth kits, and we are now very happy. (Woman, Kinesi FGD)

## References

[1] Alkema L Chou D Hogan D, et al Global, regional, and national levels and trends in maternal mortality between 1990 and 2015, with scenario-based projections to 2030: a systematic analysis by the UN Maternal Mortality Estimation Inter-Agency Group. Lancet 2016; 387: 462–474. 26584737 10.1016/S0140-6736(15)00838-7 PMC5515236

[2] UN Sustainable Development Goals. https://sustainabledevelopment.un.org (accessed 19 January 2020).

[3] Ministry of Health Community Development Gender Elderly Children (Mo-HCDGEC) [Tanzania Mainland], Ministry of Health (MoH) [Zanzibar], National Bureau of Statistics (NBS), Office of the Chief Government Statistician (OCGS), ICF. Tanzania Demographic and Health Survey and Malaria Indicator Survey (TDHS-MIS) 2015–2016, 2016 Dar es Salaam, Tanzania; Rockville, MD: Mo-HCDGEC, MOH, NBS, OCGS, and ICF.

[4] Thaddeus S Maine D. Too far to walk – maternal mortality in context. Soc Sci Med 1994; 38: 1091–1110. 8042057 10.1016/0277-9536(94)90226-7

[5] Webber G Chirangi B Magatti N. Community member and policy maker priorities in improving maternal health in rural Tanzania. Int J Gynecol Obstet 2018; 5: 1. 10.1002/ijgo.12435 29315557

[6] Fonn S Xaba M. Health workers for change: a manual to improve quality of care. Geneva: UNWP, World Bank, WHO Special Project for Research and Training in Tropical Diseases and Women’s Health Project, 1995 http://apps.who.int/iris/bitstream/10665/63192/1/TDR_GEN_95.2.pdf (accessed 21 January 2020).

[7] Webber G Chirangi B Magatti N. Promoting respectful maternity care in rural Tanzania: nurses’ experiences of the ‘Health Workers for Change’ program. BMC Health Serv Res 2018; 18: 658. 30134890 10.1186/s12913-018-3463-5 PMC6106895

[8] Bohren MA Hunter EC Munthe-Kaas HM, et al Facilitators and barriers to facility-based delivery in low- and middle-income countries: a qualitative evidence synthesis. Reprod Health 2014; 11: 71. 25238684 10.1186/1742-4755-11-71 PMC4247708

[9] Gelao A Chojenta C Musa A, et al Barriers to access and utilization of emergency obstetric care at health facilities in sub-Saharan Africa: a systematic review of literature. Syst Rev 2018; 7: 183. 30424808 10.1186/s13643-018-0842-2 PMC6234634

[10] Moshi F Nyamhanga T. Understanding the preference for homebirth: an exploration of key barriers to facility delivery in rural Tanzania. Reprod Health 2017; 14(1): 132. 29041972 10.1186/s12978-017-0397-z PMC5645928

[11] August F Pembe AB Mpembeni R, et al Community health workers can improve male involvement in maternal health: evidence from rural Tanzania. Glob Health Action 2016; 9: 30064. 26790461 10.3402/gha.v9.30064 PMC4720685

[12] Greenspan JA Chebet JJ Mpembeni R, et al Men’s roles in care seeking for maternal and newborn health: a qualitative study applying the three delays model to male involvement in Morogoro Region, Tanzania. BMC Pregnancy Childbirth 2019; 19(1): 293. 31409278 10.1186/s12884-019-2439-8 PMC6693212

[13] Obare F Warren C Abuya T, et al Assessing the population-level impact of vouchers on access to health facility delivery for women in Kenya. Soc Sci Med 2014; 102: 183–189. 24565156 10.1016/j.socscimed.2013.12.007

[14] Braun R Catalani C Wimbush J, et al Community health workers and mobile technology: a systematic review of the literature. PLoS ONE 2013; 8(6): e65772. 23776544 10.1371/journal.pone.0065772 PMC3680423

[15] Lund S Hemed M Nielsen BB, et al Mobile phones as a health communication tool to improve skilled attendance at delivery in Zanzibar: a cluster-randomised controlled trial. BJOG 2012; 119: 1256–1264. 22805598 10.1111/j.1471-0528.2012.03413.x

[16] Braun R Lasway C Agarwal S, et al An evaluation of a family planning mobile job aid for community health workers in Tanzania. Contraception 2016; 94: 27–33. 27039033 10.1016/j.contraception.2016.03.016

[17] Hackett K Lafleur C Nyella P, et al Impact of smartphone- assisted prenatal home visits on women’s use of facility delivery: results from a cluster-randomized trial in rural Tanzania. PLoS ONE 2018; 13(6): e0199400. 29912954 10.1371/journal.pone.0199400 PMC6005474

[18] Mwendwa P. Assessing the fit of RapidSMS for maternal and newborn health: perspectives of community health workers in rural Rwanda. Develop Prac 2016; 261: 38–51.

[19] Webber G Chirangi B Magatti N. Challenges and successes of distributing birth kits with misoprostol to reduce maternal mortality in rural Tanzania. Afr J Reprod Health 2019; 23(3): 78–168. 10.29063/ajrh2019/v23i3.7 31782633

[20] Bohren MA Vogel JP Hunter EC, et al The mistreatment of women during childbirth in health facilities globally: a mixed-methods systematic review. PLoS Med 2015; 12(6): e1001847. 26126110 10.1371/journal.pmed.1001847 PMC4488322

